# Role of Anion Identity in the Assembly and Morphology of Whey Protein Isolate Nanofibril Aggregates

**DOI:** 10.3390/foods15132280

**Published:** 2026-06-25

**Authors:** Shirong Dong, Wei Xu, Yu Sun, Yuju Yang, Chun Bian, Qi Han

**Affiliations:** 1 School of Food Engineering, Harbin University, Harbin 150086, China; xweihappy@163.com (W.X.); sunyu87926@126.com (Y.S.); ji3526183@126.com (C.B.); 2 College of Food, Heilongjiang Bayi Agricultural University, Daqing 163319, China; hqneau@163.com

**Keywords:** whey protein isolate, polymerization kinetics, metal ions, fibril aggregates, anion

## Abstract

To investigate the respective contributions of cations (Na^+^, K^+^, Zn^2+^, Fe^3+^) and anions (Cl^−^ and SO_4_^2−^) to the formation of whey protein isolate (WPI)-based nanofibrils, eight salts with a 4 × 2 factorial design were added to WPI solutions. The morphology and aggregation process of the fibril aggregates were examined under fixed low salt concentration (10 mmol/L) to isolate ion-specific effects. The salts altered the pH, conductivity, and fibril yield. Notably, the Na^+^, K^+^, and Zn^2+^ salts increased fibril production, whereas Fe^3+^ salts reduced it. Mechanistically, Fe^3+^ strongly suppressed fibrillation via strong electrostatic interaction and accelerated protein hydrolysis, while SO_4_^2−^ partially alleviated this inhibition. All the ions altered the kinetic parameters. Compared with Cl^−^ salts, SO_4_^2−^ salts induced shorter, clustered fibrils and stronger kinetic suppression, preserving elongated fibrils. X-ray photoelectron spectroscopy (XPS) confirmed anion incorporation, and X-ray diffraction (XRD) revealed secondary structural changes. These results demonstrate that while cations contribute to fibril formation, anions play a deterministic role in regulating assembly kinetics and morphological outcomes, independent of cation valence. In this study, we establish a mechanistic basis for tailoring WPI fibril aggregation states through anion-specific salt selection.

## 1. Introduction

Almost all the proteins derived from sources such as dairy, eggs, rice, soy, peas and lentils can be assembled into protein fibril aggregates [1] because they contain at least one short sequence capable of generating amyloid fibrils [2]. Compared with native protein, fibril aggregates have a high aspect ratio and better surface activity and stability [3]. Fibril formation confers novel functionalities on native proteins. Fibrillization kinetics, aggregate morphology, and final structure are critically modulated by multiple factors, such as protein concentration, ions, temperature, pH, agitation, and salt type [4,5,6,7,8]. Controlling the ions present is a simple and convenient method of regulating the formation of fibrils because ions are readily available and inexpensive. The morphology and formation kinetics of rice protein fibrils can be regulated by the metal ions Fe^3+^, Cu^2+^, and Ca^2+^ [5]. Moreover, the fibril kinetics of rice glutelin were accelerated in the presence of Hofmeister series cations (NH_4_^+^, K^+^, Na^+^, Li^+^, Mg^2+^, Ca^2+^, Zn^2+^, Cu^2+^) [9]. Mijin et al. [10] reported that Pb^2+^ and Cd^2+^ affect the morphology of ovalbumin fibrils by increasing fibril branching and forming fibril clusters, possibly due to their different binding sites on ovalbumin. Since Cl^−^ is in the middle of the Hofmeister series (lyotropic sequences or ion specificity) of anions, it neither makes nor breaks the water structure [11]. Most studies in this area have investigated chloride salts as the research subject. However, in practical applications, more complex salts, such as sulfate (SO_4_^2−^) salts, are also frequently used. Importantly, research on the effects of sulfate salts on fibril aggregates is relatively limited.

Ionic strength has a pronounced concentration-dependent influence on fibrillation kinetics and the resulting fibril morphology (e.g., length, flexibility, and entanglement) [12,13,14]. Added ions mediate electrostatic screening, attenuating intermolecular repulsion to modulate both fibrillation pathways and supramolecular architecture [15]. Similarly, elevated NaCl concentrations during the formation of soy protein isolate (SPI) fibrils diminish electrostatic repulsion, yielding elongated flexible fibrils [16]. Ionic strength modulates electrostatic interactions among fibril constituents. Key mechanisms involve cation bridging between carboxylate groups (aspartic/glutamic acid side chains, C-termini) and cation-facilitated monomer binding, collectively modulating fibrillation kinetics along with the structural and rheological properties of protein nanofibrils [16]. Divalent cations (Mg^2+^, Ca^2+^, Ba^2+^) typically accelerate formation kinetics more effectively than monovalent ions (Li^+^, Na^+^, K^+^), with minimal variation observed within the same valence group [17]. The kinetic, rheological, and morphological effects of group IA and IIA cations on whey protein nanofibrils have been systematically characterized by Loveday et al. [17]. Although salt valence influences the dimensions and connectivity of nanofibril networks (accounting for bulk rheological differences), individual fibril morphology generally remains unaffected. However, excessive salt concentrations induce charge shielding, which impairs the hydrophobic interactions that are essential for nucleation, promoting the formation of amorphous aggregates over ordered nanofibrils [15,18]. Most existing studies have focused primarily on relatively high salt ion concentrations, particularly using chloride (Cl^−^) salts. In real-world actual food processing, however, the types of salt ions used are more complex, and the concentrations are relatively lower. At the same time, research on the effects of sulfate salts on fibril aggregates is comparatively limited, even though recent studies on other food proteins have highlighted the crucial role of anions [19]. A comprehensive review by Cheng et al. [20] also pointed out that most fibrillation studies focus on chloride salts, leaving the effects of sulfate salts underexplored. The specific effects of anions on protein fibrillation in acidic environments are known to follow the Hofmeister series, as demonstrated in lysozyme systems [21,22]. Moreover, recent studies on wheat gluten have shown that NaCl concentration critically modulates fibril morphology [23].

Whey protein isolate (WPI), a primary by-product of cheese production, possesses outstanding nutritional properties and diverse functional characteristics. After fibrillation, WPI-derived fibril proteins exhibit promising application prospects as thickeners, Pickering emulsion stabilizers, and carriers for bioactive nutraceutical ingredients. Although plenty of studies have investigated the effects of salt concentration on WPI fibril formation, in practical industrial production, salt concentration is generally low, and systematic comparative studies examining the influences of chloride and sulfate ions on WPI fibrillation at low ionic concentration are still lacking [20,24,25].

To systematically dissect the individual and combined effects of cation valence and anion identity on WPI fibril assembly, a set of eight salts was designed: four cations (Na^+^, K^+^, Zn^2+^, Fe^3+^) representing monovalent, divalent, and trivalent valences, and two anions (Cl^−^ and SO_4_^2−^) with different positions in the Hofmeister series. This 4 × 2 matrix allowed us to: (i) compare the effect of chloride vs. sulfate under exactly the same cationic conditions, (ii) examine whether the influence of anions is consistent across different cations, and (iii) identify possible synergistic or antagonistic effects between specific cations and anions (e.g., Fe^3+^ with SO_4_^2−^ partially rescuing the inhibition caused by Fe^3+^ alone). A smaller set of salts would have been insufficient to separate the contributions of cation valence and anion identity, while a larger set would have been redundant. The eight salts chosen provide a balanced and systematic framework for this mechanistic study. Previous studies have investigated the effects of cations on whey protein fibrils and the role of Hofmeister anions in other protein systems [17,19,21], but the use of a systematic 4 × 2 matrix combining four cations and two anions at low salt concentration has not been reported.

Therefore, in this study, we aimed to systematically evaluate how the identity of anions (Cl^−^ vs. SO_4_^2−^), in combination with mono-, di-, and trivalent cations, modulates the fibrillation kinetics, secondary structure, and supramolecular morphology of WPI nanofibrils at low ionic strength. We evaluated eight salt combinations (NaCl, Na_2_SO_4_, KCl, K_2_SO_4_, ZnCl_2_, ZnSO_4_, FeCl_3_, and Fe_2_(SO_4_)_3_) by incubating 3% WPI solutions at 90 °C (pH 2.0) for 0–10 h and systematically characterized the fibrillation kinetics and aggregate morphology.

## 2. Materials and Methods

### 2.1. Materials

WPI was obtained from Hilmar Corporation (Hilmar Cheese, CA, USA) and had the following composition: 93% protein, 4.5% moisture, 1.3% fat, 0.6% lactose, and 3.0% ash. Thioflavin T was sourced from Sigma-Aldrich (St. Louis, MO, USA). Analytical grade salts, including sodium salts (NaCl and Na_2_SO_4_), potassium salts (KCl and K_2_SO_4_), zinc salts (ZnCl_2_ and ZnSO_4_), and iron salts (FeCl_3_ and Fe_2_(SO_4_)_3_), were obtained from commercial suppliers. HCl was also obtained from commercial suppliers.

### 2.2. Sample Preparation

A 3.5% (*w*/*v*) WPI solution in deionized water was centrifuged (12,000 rpm, 20 min), and the supernatant was collected. The protein content was quantified via Kjeldahl nitrogen analysis. This clarified solution was diluted to 3.0% (*w*/*v*) protein and adjusted to pH 2.0 using 2 mol/L and 0.2 mol/L HCl. The solution was divided into nine 100 mL aliquots: one untreated control and eight aliquots supplemented with specified salts (NaCl, Na_2_SO_4_, KCl, K_2_SO_4_, ZnCl_2_, ZnSO_4_, FeCl_3_, and Fe_2_(SO_4_)_3_) to achieve a 10 mmol/L salt concentration. All the samples were heated at 90 °C for 0, 1, 2, 3, 4, 5, 6, 7, 8, 9, and 10 h under static conditions (without stirring) [26,27]. After immediate cooling, the solutions were stored at 4 °C for fibril maturation. The selection of these eight salts (Table 1) enabled us to carry out a full factorial comparison of four cations (Na^+^, K^+^, Zn^2+^, Fe^3+^) and two anions (Cl^−^, SO_4_^2−^) at a fixed low salt concentration (10 mmol/L). The sample designations are detailed in Table 1. For the 10 h heated samples, portions were preserved as liquids or freeze-dried for analysis.

### 2.3. Visual Image Acquisition

Digital images of WPI solutions (with and without added NaCl, Na_2_SO_4_, KCl, K_2_SO_4_, ZnCl_2_, ZnSO_4_, FeCl_3,_ or Fe_2_(SO_4_)_3_) were captured after heating at 90 °C for 0–10 h at hourly intervals.

### 2.4. Determination of Turbidity

After overnight storage at 4 °C, the turbidity was measured using a UV spectrophotometer (TU-1800, Beijing Precision Instruments Co., Ltd., Beijing, China) at 500 nm using deionized water as a blank. This wavelength was chosen to avoid interference from protein absorption maxima (typically approximately 280 nm) while remaining sensitive to light scattering from protein aggregates and fibrils, as previously described in studies of protein fibrillation studies [28]. The absorbance values for all the solutions (WPI, NaCl-WPI, Na_2_SO_4_-WPI, KCl-WPI, K_2_SO_4_-WPI, ZnCl_2_-WPI, ZnSO_4_-WPI, FeCl_3_-WPI, and Fe_2_(SO_4_)_3_-WPI) were recorded after 0–10 h heating intervals.

### 2.5. Determination of pH

The pH values of WPI solutions and salt-supplemented WPI solutions (NaCl-WPI, Na_2_SO_4_-WPI, KCl-WPI, K_2_SO_4_-WPI, ZnCl_2_-WPI, ZnSO_4_-WPI, FeCl_3_-WPI, and Fe_2_(SO_4_)_3_-WPI) were measured after heating for 0–10 h. Measurements were performed using a calibrated benchtop pH meter (FE28, Mettler-Toledo, Shanghai, China) equipped with a standard glass combination electrode [29].

### 2.6. Determination of Conductivity

A conductivity meter (DDSJ-318T Conductivity Meter Shanghai Yidian Science Instrument Co., Ltd., Shanghai, China) was used to measure the conductivity of the sample.

### 2.7. TEM Analysis

A 3 μL aliquot of each diluted suspension (WPIF, NaCl-WPIF, Na_2_SO_4_-WPIF, KCl-WPIF, K_2_SO_4_-WPIF, ZnCl_2_-WPIF, ZnSO_4_-WPIF, FeCl_3_-WPIF, and Fe_2_(SO_4_)_3_-WPIF) was deposited on carbon-coated copper grids for 15 min. Excess liquid was removed via filter paper wicking [16]. No negative staining (e.g., uranyl acetate or phosphotungstic acid) was applied to avoid potential artifacts that might alter fibril morphology. Air-dried samples were examined using a Hitachi H-7650 transmission electron microscope (H-7650 Hitachi High Technologies Corp.) operated at 100 kV.

### 2.8. ThT Fluorescence Intensity Determination

A 3.0 mmol/L ThT stock solution in phosphate/NaCl buffer (10 mM phosphate, 150 mM NaCl, pH 7.0) was filter-sterilized (0.2 µm Millex-GS) and stored at 4 °C in light-protected vials. Working solutions (60 µM ThT) were prepared by 50-fold dilution of stock in fresh buffer. For analysis, 96 µL aliquots of samples were combined with 8 mL of ThT working solution, mixed via vortexing, and incubated at room temperature (1 min). Fluorescence was measured using a Hitachi F7100 spectrometer (Hitachi High-Technologies Corp., Tokyo, Japan) with spectral parameters set at λ ex 460 nm/λ em> 490 nm and 10 nm slit width [6].

### 2.9. Polymerization Kinetics of Whey Protein Nanofibrils

Thioflavin T fluorescence data were fitted to the empirical equation (Equation (1)) proposed by Morris et al. [30], where *f*_t_ represents the fluorescence intensity at time t, and α, β, and γ are fitting parameters.

(1) $$f_{t}=\gamma -\frac{\gamma +\frac{\beta }{\alpha }}{1+\frac{\beta }{\alpha \;\gamma }\exp[(\beta +\alpha \;\gamma )]}$$

(2) $$t_{lag}=\frac{1}{\beta +\alpha \;\gamma }[\ln(\frac{\alpha \;\gamma }{\beta })-4\frac{\alpha \;\gamma }{\beta +\alpha \;\gamma }+2]$$

(3) $$t_{1/2max}=\frac{\ln(2+\frac{\alpha \;\gamma }{\beta })}{\beta +\alpha \;\gamma }$$

(4) $$(\frac{df}{dt}{)}_{max}=\frac{\left(\frac{\beta }{\gamma }+\alpha )(\beta +\alpha \;\gamma \right)}{4}$$

The kinetic parameters were lag time (*t*_lag_), time to reach half-maximal fluorescence (*t*_1/2max_), and maximum fluorescence increase rate (*df*/*dt*)_max,_ and they were calculated using the analytical expressions derived from the model equation [Equation (1)] in earlier research (Equations (2)–(4)) [6].

### 2.10. XPS Determination

To establish baseline levels of Cl and S originating from the sample preparation procedure (pH adjusted with HCl) and from intrinsic components of WPI (e.g., sulfur-containing amino acids, trace minerals in ash), a control sample of WPI fibrils formed without any salt addition (native WPIF) was prepared and analyzed under identical XPS conditions. The elemental signals obtained from this control sample were used as background references for the salt-supplemented samples. The elemental compositions (Cl^−^, SO_4_^2−^, Na^+^, K^+^, Zn^2+^, and Fe^3+^) of the native WPIF, NaCl-WPIF, Na_2_SO_4_-WPIF, KCl-WPIF, K_2_SO_4_-WPIF, ZnCl_2_-WPIF, ZnSO_4_-WPIF, FeCl_3_-WPIF, and Fe_2_(SO_4_)_3_-WPIF samples were analyzed via XPS [31]. Samples were prepared in 10 mM solutions of their respective salts (NaCl, Na_2_SO_4_, KCl, K_2_SO_4_, ZnCl_2_, ZnSO_4_, FeCl_3_, and Fe_2_(SO_4_)_3_). High-resolution spectra were processed using 2021 Origin software to quantify the elemental peak areas (e.g., Cl^−^, SO_4_^2−^, Na^+^, K^+^, Zn^2+^, and Fe^3+^). The peak area of each element directly correlated with its surface concentration bound to fibrils, enabling comparative analysis of elemental binding across samples.

### 2.11. CD Determination

Secondary structure transitions in the WPI, WPIF, NaCl-WPIF, Na_2_SO_4_-WPIF, KCl-WPIF, K_2_SO_4_-WPIF, ZnCl_2_-WPIF, ZnSO_4_-WPIF, FeCl_3_-WPIF, and Fe_2_(SO_4_)_3_-WPIF samples were characterized via circular dichroism spectroscopy (Jasco J-815, Tokyo, Japan) [32]. The samples were diluted to 0.25 mg/mL for spectral acquisition between 190 and 260 nm. Three successive scans were averaged per sample. Quantitative secondary structure analysis was performed using followed by quantitative analysis of secondary structure content with CDNN software (Version 4.0, Gerald Böhm, Bioinformatics, Germany). 

### 2.12. XRD Determination

X-ray diffraction analysis was performed on freeze-dried samples (control and ion-modified WPIF) using a Rigaku UltimaIV diffractometer (Rigaku Corporation, Tokyo, Japan) with Cu-Kα radiation (40 kV/40 mA). The scans covered a 5–50° 2θ range at 0.05° step intervals and a 1°/s scan rate. XRD patterns were processed using MDI Jade 9 software.

### 2.13. Statistical Analysis

All analyses were conducted in triplicate. Statistical significance (*p* < 0.05) was determined via one-way ANOVA using Statistix 8.0, with data visualization performed using Origin 2021 (OriginLab Corporation, Northampton, MA, USA).

## 3. Results

### 3.1. Analysis of Visual Diagrams

**Figure 1:**
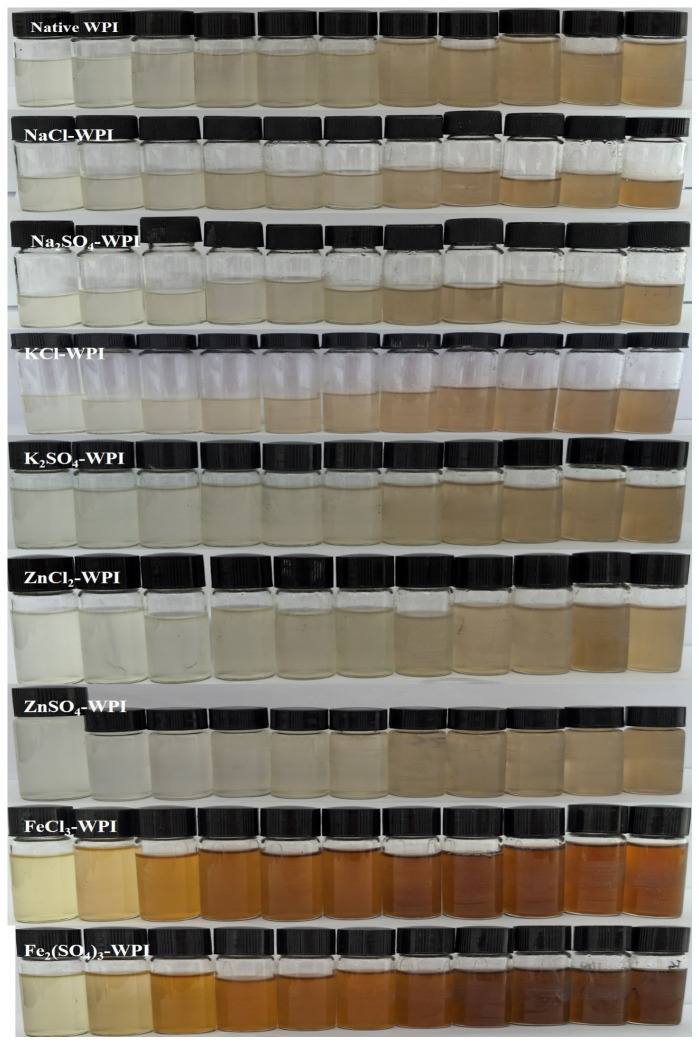
The visual appearances of WPI solutions with different salts (NaCl, Na_2_SO_4_, KCl, K_2_SO_4_, ZnCl_2_, ZnSO_4_, FeCl_3_, Fe_2_(SO_4_)_3_) during heating at 90 °C for 0–10 h. Darkening increases with heating time; Fe^3+^-containing solutions rapidly turn reddish black, while sulfate-containing solutions generally show lighter coloration than chloride-containing ones under the same cation.

Eight salts were chosen to systematically evaluate the effects of cation valence and anion identity. The results are presented below and follow this 4 × 2 design. Eight chloride/sulfate salts (NaCl, Na_2_SO_4_, KCl, K_2_SO_4_, ZnCl_2_, ZnSO_4_, FeCl_3_, and Fe_2_(SO_4_)_3_) were added to 3% WPI solutions (10 mmol/L salt concentration) and heated at 90 °C for 0–10 h. Figure 1 provides a qualitative visual overview of the samples. Notable differences were observed, such as the rapid darkening to reddish-black hues in Fe^3+^-containing solutions. Quantitative assessments of aggregate formation are presented in the following sections (turbidity, ThT fluorescence, etc.).

### 3.2. Turbidity and pH Change Analysis

**Figure 2:**
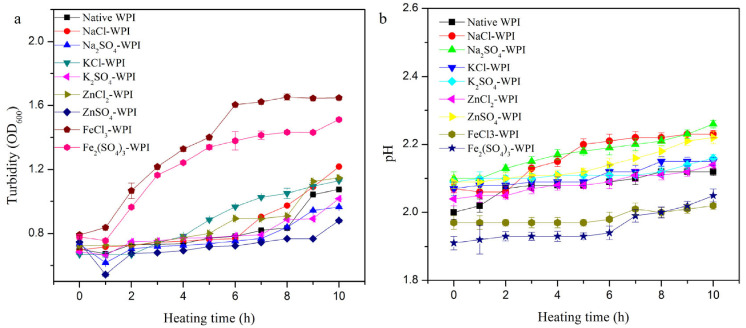
The turbidity (**a**) and pH (**b**) of the WPI solutions with different salts (NaCl, Na_2_SO_4_, KCl, K_2_SO_4_, ZnCl_2_, ZnSO_4_, FeCl_3_, Fe_2_(SO_4_)_3_) during heating at 90 °C for 0–10 h.

The turbidity changes in all tested WPI solutions, heated at 90 °C for 0–10 h, are shown in Figure 2a. During heating, the turbidity of native WPI increased from 0.70 to 1.07, reflecting the hydrolysis of proteins into smaller peptides followed by their assembly into larger aggregates [33,34]. For chloride salts (NaCl, KCl and ZnCl_2_), turbidity increased steadily throughout heating. In contrast, all sulfate salts (Na_2_SO_4_, K_2_SO_4_, and ZnSO_4_) exhibited an initial decrease followed by a subsequent increase. Under the same cationic conditions, the final turbidities of sulfate-containing samples were consistently lower than those of the corresponding chloride-containing samples, suggesting that sulfate ions promote the formation of smaller or more compact aggregates during the early stage of heating. This is consistent with the accelerated nucleation kinetics (shorter lag time, Table 2), indicating that SO_4_^2−^ favors rapid nucleation over lateral growth [22].

After FeCl_3_ was added, the turbidity increased from 0.79 to 1.65 during heating. After Fe_2_(SO_4_)_3_ was added, the turbidity first decreased from 0.78 to 0.76 and then increased to 1.51. The distinct turbidity behavior of Fe^3+^-containing solutions, along with the rapid darkening observed in Figure 1, may arise from two combined effects. First, Fe^3+^ can hydrolyze under acidic heating conditions to form colloidal ferric hydroxides or precipitates, contributing to turbidity and color changes [34]. Second, Fe^3+^, as a trivalent cation, exhibits strong electrostatic interactions with negatively charged protein residues, which may promote protein aggregation and precipitation rather than ordered fibrillation [5]. The less pronounced turbidity increase in Fe_2_(SO_4_)_3_ compared with FeCl_3_ may be attributed to the complexation between SO_4_^2−^ and Fe^3+^, which partially reduces the effective concentration of free Fe^3+^ and alleviates its precipitation-inducing effect. Hydrolysis is essential for fibril formation; excessively rapid aggregation is unfavorable for fibril development [33,34].

The pH changes of all tested WPI solutions during the 10 h incubation are shown in Figure 2b. pH was monitored because protein fibrillation under acidic conditions involves peptide bond hydrolysis, which releases free amino groups and consumes H^+^, leading to a gradual pH increase. Thus, the pH rise serves as an indirect indicator of fibril formation and hydrolysis [29,35]. Upon salt addition, monovalent (Na^+^, K^+^) and divalent (Zn^2+^) salts immediately raised the pH, with sulfate salts causing a larger increase than chloride salts under the same cation. This is attributed to sulfate’s ability to complex metal ions, suppressing their hydrolysis and reducing H^+^ concentration. In contrast, Fe^3+^ salts immediately lowered the pH, with Fe_2_(SO_4_)_3_ causing a more significant reduction than FeCl_3_. During heating, pH increased continuously for almost all samples (except Fe^3+^ modified samples), consistent with fibril formation and peptide bond hydrolysis [15,36]. Ye et al. [29] also reported a relationship between pH increase and prolonged heating time.

The initial pH differences upon adding different metal ions arise from the varying acidity of these ions, which depends on their acid dissociation constants (pKa values). The pKa values of K^+^, Na^+^, and Fe^3+^ are 14.5, 14.2, and 2.2, respectively [37]. Therefore, cations with different valences alter the initial pH of WPI solutions to varying extents.

### 3.3. Changes in Conductivity

**Figure 3:**
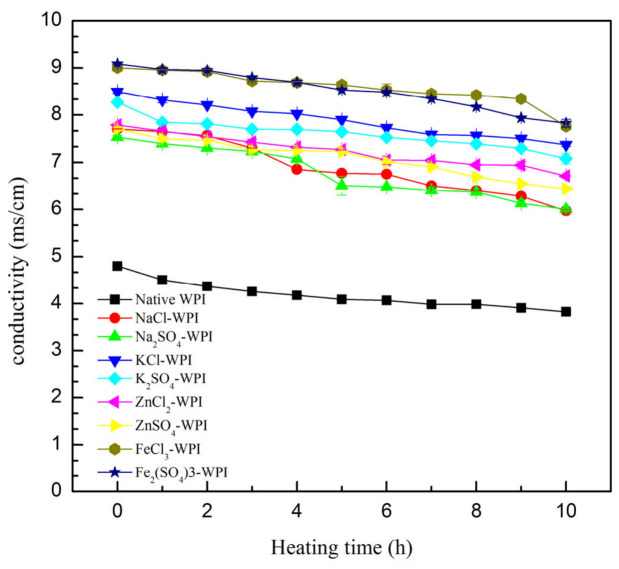
Conductivity of the WPI solutions with different salts (NaCl, Na_2_SO_4_, KCl, K_2_SO_4_, ZnCl_2_, ZnSO_4_, FeCl_3_, Fe_2_(SO_4_)_3_) during heating at 90 °C for 0–10 h.

Conductivity was monitored because the binding of salt ions (cations and anions) to fibrils or their incorporation into aggregates reduces the concentration of free ions in solution, decreasing conductivity over time. Conductivity changes therefore reflect ion–fibril interactions and aggregation dynamics. Changes in the conductivity of all tested WPI solutions during heating at 90 °C for 0–10 h are shown in Figure 3. During the heating process, the conductivities of all the samples tended to decrease, but the magnitude of the decrease varied. The conductivity changes for all tested WPI solutions during heating are shown in Figure 3. For most salts (monovalent and divalent chlorides and sulfates), conductivity decreased during heating, with the extent of decrease varying by salt type. In contrast, Fe^3+^ salts uniquely caused an increase in conductivity, with Fe_2_(SO_4_)_3_ showing a slightly larger increase than FeCl_3_.

In contrast, the conductivity of Fe^3+^-containing solutions uniquely increased during heating, reaching 9.00 mS/cm for FeCl_3_ and 9.08 mS/cm for Fe_2_(SO_4_)_3_ after 10 h. respectively, after the addition of FeCl_3_ and Fe_2_(SO_4_)_3_. For the other salts, the final conductivities after 10 h heating ranged from 56% to 93% of their initial values, whereas Fe^3+^ salts exceeded their initial conductivities (102–105%). These results indicate that the addition of different salts altered both the initial conductivity and its evolution during heating, with Fe^3+^ salts showing a distinct increasing trend.

### 3.4. Effects of Different Salts on the ThT Intensity and Self-Assembly Kinetics

**Figure 4:**
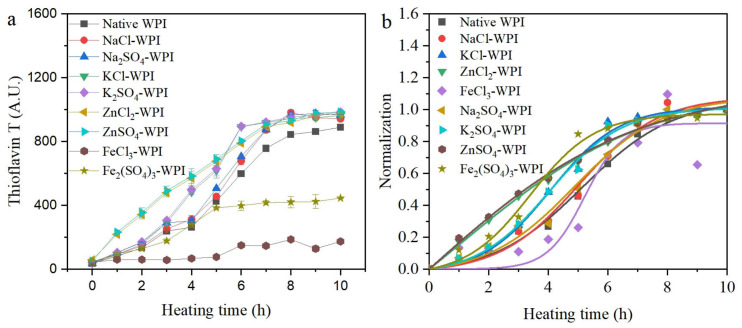
Thioflavin T fluorescence (**a**) and fitting results of thioflavin T fluorescence (**b**) for native WPI and NaCl-WPI, Na_2_SO_4_-WPI, KCl-WPI, K_2_SO_4_-WPI, ZnCl_2_-WPI, ZnSO_4_-WPI, FeCl_3_-WPI, and Fe_2_(SO_4_)_3_-WPI solutions heated for 10 h at 90 °C.

The ThT fluorescence assay is a sensitive and effective technique for detecting the formation of fibrils because ThT dyes can specifically bind to the cross-β structure of fibril aggregates [9]. The fibrillation yield and β-sheet amount were closely related to the intensity of ThT fluorescence [4]. The changes in ThT fluorescence intensity during heating at 90 °C (0–10 h) for all tested WPI solutions are shown in Figure 4a. All systems exhibited increasing fluorescence, albeit with distinct kinetic profiles [3]. Native WPI displayed characteristic sigmoidal growth. For monovalent cations (Na^+^ and K^+^), the ThT fluorescence intensity increased substantially during heating, reaching similar high maximum values regardless of the accompanying anion, indicating efficient fibril formation. The divalent Zn^2+^ systems also showed marked increases in fluorescence intensity, suggesting that higher ionic valency improves fibril yield, which agrees with Li et al. [9].

In contrast, Fe^3+^ systems exhibited severely suppressed, non-sigmoidal kinetics, with much lower maximum fluorescence intensities, indicating that Fe^3+^ inhibits WPI fibril formation, consistent with Qi et al. [5]. Moreover, the inhibitory effects were anion-dependent. Among the anions tested, the presence of SO_4_^2−^ partially alleviated the suppression compared to Cl^−^.

The aggregation kinetics of WPI in the presence of all tested salts were evaluated using ThT fluorescence. The normalized ThT fluorescence data were fitted to the empirical function proposed by Morris et al. [31], with the fitted curves depicted in Figure 4b and the resulting kinetic parameters summarized in Table 2. The addition of salt significantly influenced the fibrillation kinetics of WPI (*p* < 0.05) [38]. High fitting accuracy was achieved for all the systems(R^2^ > 0.85, Table 2), confirming the reliability of the kinetic analysis, although the FeCl_3_-WPI system exhibited a relatively lower R^2^ (0.85) due to severe suppression of fibrillation by Fe^3+^, which deviates from the sigmoidal assumption of the Morris model. As summarized in Table 2, monovalent (Na^+^, K^+^) and divalent (Zn^2+^) salts resulted in similarly high maximum ThT fluorescence intensities (f_max_ = 962–985 A.U.), indicating efficient fibril formation. In contrast, Fe^3+^ salts severely reduced the fluorescence yield, with FeCl_3_ showing the strongest inhibition (f_max_ = 173 A.U.), while partial recovery was observed with Fe_2_(SO_4_)_3_ (f_max_ = 445 A.U.). All differences were statistically significant (*p* < 0.05). These results demonstrate that metal ions significantly affect the formation of WPI fibril aggregates.

Additionally, the lag time (t_lag_), half-maximum time (t_1/2max_), and maximum rate of change (df/dt)_max_ of the WPI were influenced by the addition of metal ions. The lag times (t_lag_) derived from kinetic fitting are summarized in Table 2. Compared with native WPI (t_lag = 2.308 h), monovalent salts (Na^+^ and K^+^) gave comparable or slightly shorter lag times, while divalent Zn^2+^ salts dramatically reduced t_lag_ to near zero; such extremely short lag times are reminiscent of homologous seeding, where preformed fibril fragments act as nuclei to bypass the slow nucleation phase [39]. In contrast, FeCl_3_ prolonged the lag time to 3.972 h, but Fe_2_(SO_4_)_3_ shortened it to 1.414 h, suggesting that SO_4_^2−^ partially alleviated the inhibitory effect of Fe^3+^.

Notably, the kinetic fitting results (Table 2) revealed that sulfate-containing salts consistently yielded shorter lag times than their chloride-containing counterparts under the same cationic conditions. According to previous studies, salts can improve the assembly of heat-induced protein fibrils at pH 2.0 by shortening the lag phase and increasing the fibril growth rate [38,40]. The formation of fibril aggregates from WPI involves an initial hydrolysis step followed by nucleation, and the addition of metal ions may affect the hydrolysis rate and nucleation process, thereby influencing the kinetic parameters [34].

Overall, the kinetic parameters demonstrated that different metal ions can significantly influence the polymerization kinetics of WPI and alter the maximum ThT fluorescence intensity. Furthermore, protein fibrillation is regulated not only via the salting-out effect but also via electrostatic interactions the complex interplay in Debye–Hückel screening, ion selectivity, and the Hofmeister effect [9,41,42].

### 3.5. Effects of Different Salts on Morphologies

**Figure 5:**
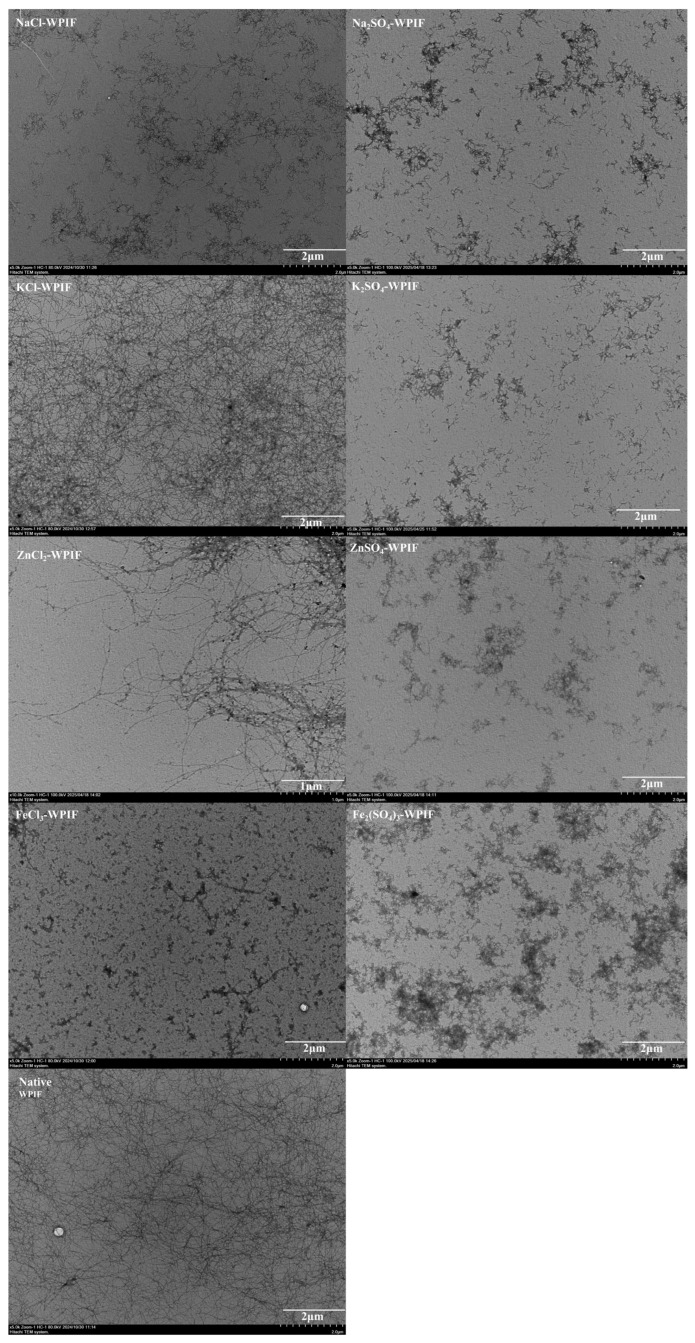
TEM micrographs of native WPIF, NaCl-WPIF, Na_2_SO_4_-WPIF, KCl-WPIF, K_2_SO_4_-WPIF, ZnCl_2_-WPIF, ZnSO_4_-WPIF, FeCl_3_-WPIF, and Fe_2_(SO_4_)_3_-WPIF. The scale bar indicates 1 μm.

The morphologies of all tested WPI are shown in Figure 5. Control WPIF (without added ions) exhibited long, straight fibrils. NaCl-WPIF maintained fibril elongation but introduced bent conformations, which is consistent with prior reports [17,36]. At low Na_2_SO_4_ concentrations (10 mM), two distinct fibril types coexisted: elongated fibrils and short, tightly curled, ‘worm-like’ structures. KCl preserved the native-like slender fibrils, whereas K_2_SO_4_ promoted compact helical winding. ZnCl_2_ facilitated extended straight fibrillization, whereas ZnSO_4_ yielded smaller, clustered fibrillar aggregates with increased curvature. FeCl_3_ generated hypertrophied fibrils (increased length and diameter) with surface-adherent amorphous aggregates (Figure 3). Fe_2_(SO_4_)_3_ caused severe structural collapse, producing short-branched polymers embedded within dense clusters. The distinct effects of Cl^−^ vs. SO_4_^2−^ counterions suggest an anion-specific modulation of assembly pathways. This morphological divergence likely stems from differences in metal ion solvation capacities: the stronger hydration shells associated with SO_4_^2−^-coordinated ions may accelerate proteolytic cleavage, favoring rapid nucleation over elongation [31]. Consistent with this mechanism, the influence of SO_4_^2−^ on the aggregation process was more pronounced than that of Cl^−^. These results demonstrate that salt composition critically regulates fibril architecture.

### 3.6. XPS Spectral Analysis

**Figure 6:**
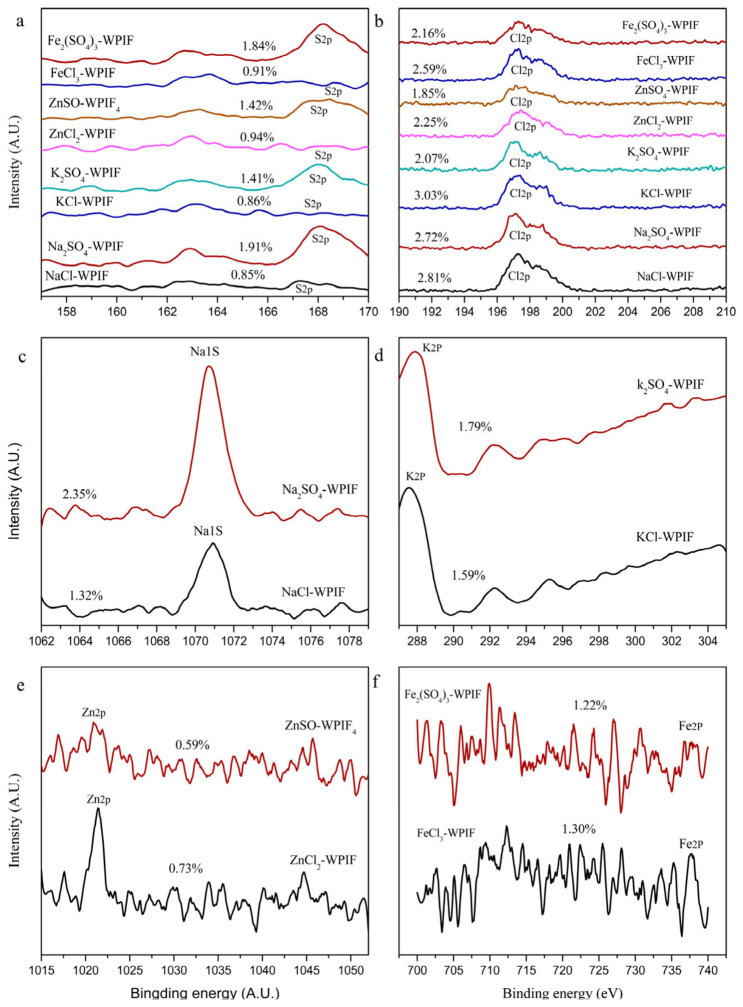
X-ray photoelectron survey spectra of salt-free WPIF control and WPIF samples formed in the presence of 10 mM of all tested salts after incubation at 90 °C for 10 h. All samples were prepared under identical conditions (pH 2.0 adjusted with HCl). The salt-free WPIF control was used to establish background Cl and S levels from sample preparation and intrinsic WPI composition. Panels (**a**–**f**) show signals for Cl, S, Na, K, Zn, and Fe, respectively.

X-ray photoelectron spectroscopy (XPS) was performed to probe the surface elemental composition of the fibril samples. To distinguish between signals arising from the added salts and those originating from the sample preparation or WPI itself, a salt-free WPIF control sample was analyzed in parallel. The results are presented in Figure 6. The binding quantities were determined from the elemental peak areas, which reflect the salt composition of the fibrils [42]. The percentages in Figure 4 represent the normalized peak area of each element relative to the total number of detectable elements within the sample. A Cl signal was detected in all samples, including the salt-free control, which is attributed to residual Cl^−^ from the HCl used to adjust the solution to pH 2.0 prior to heating. However, salt-supplemented samples generally exhibited higher Cl signals than the control. For example, NaCl-WPIF and KCl-WPIF showed Cl signals approximately twice that of the control, indicating additional Cl^−^ binding from the added chloride salts. FeCl_3_-WPIF gave the highest Cl signal relative to the control, consistent with the strong inhibitory effect of Fe^3+^ on fibrillation. A S signal was also present in all samples, including chloride-only samples and the salt-free control. This intrinsic S originates from sulfur-containing amino acids (methionine and cysteine) in WPI, as well as possible trace sulfate from the ash fraction. For sulfate-supplemented samples, the S signal was consistently higher than those of the salt-free control and their chloride-only counterparts. This elevated S is attributed to the incorporation of additional sulfur from the added sulfate salts. Signals corresponding to the respective cations (Na^+^, K^+^, Zn^2+^, Fe^3+^) were detected exclusively in the salt-supplemented samples, with negligible signals in the control or mismatched samples, confirming the specific binding of these cations to the fibril surface. These results reaffirm that both anions and cations associate with WPI fibrils, with the extent of association depending on the specific salt identity. The combined effects of Fe^3+^ and SO_4_^2−^ drive structural changes. Crucially, compared with Cl^−^, SO_4_^2−^ consistently exerted a stronger influence on aggregation, and these results were consistent with the prior results [8,36]. Teng et al. [3] also reported that anions have a more pronounced effect than cations on the aggregation power of proteins. Collectively, these findings demonstrate that ionic valence modulates fibrillation efficacy, with higher-valence ions (e.g., SO_4_^2−^ vs. Cl^−^; Fe^3+^ vs. Na^+^) having a greater effect on protein assembly pathways.

### 3.7. CD and XRD Analysis

**Figure 7:**
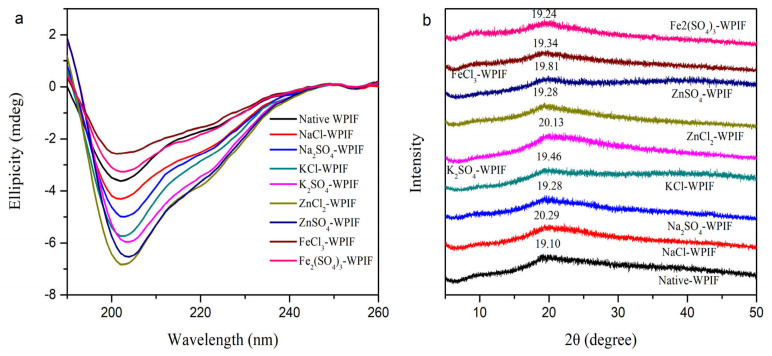
Changes in the circular dichroism (**a**) and XRD spectra (**b**) of NaCl-WPIF, Na_2_SO_4_-WPIF, KCl-WPIF, K_2_SO_4_-WPIF, ZnCl_2_-WPIF, ZnSO_4_-WPIF, FeCl_3_-WPIF, and Fe_2_(SO_4_)_3_-WPIF.

We first clarify the spectral features corresponding to different secondary structures in the CD spectra. In the far-UV CD region (190–260 nm), a negative peak near 208–222 nm indicates α-helix, a negative peak near 215–220 nm represents β-sheet, a peak around 200–210 nm corresponds to β-turn, and values below 200 nm or weak broad signals reflect random coil. These characteristic positions were used to calculate the percentages of α-helix, β-sheet, β-turn, and random coil, as listed in Table 3. Far-ultraviolet circular dichroism (far-UV CD) spectroscopy (190–260 nm) provides a sensitive probe for monitoring secondary structural transitions during protein fibrillation [3]. The CD spectra of the native WPI and salt-treated WPI solutions after heating for 10 h at 90 °C are compared in Figure 7a. Quantitative secondary structure analysis (Table 3) revealed significant variations in β-sheet content across samples. A pronounced blueshift in the minimum ellipticity following fibrillation indicates the disruption of the native secondary structure and the release of short peptides under acidic heating conditions [43]. Native WPI had 28.70% β-sheet content, which is consistent with the value reported by Yang et al. (27.3%) [31]. In contrast, the β-sheet content increased to 37.10% in the control WPIF (no added salt), confirming that fibrillation promotes the formation of ordered β-sheet-rich structures essential for WPNF formation [3,29]. Far-UV CD spectra (Figure 7a) and quantitative secondary structure analysis (Table 3) revealed that all salts except Fe^3+^ increased β-sheet content relative to native WPI (28.7%). The highest β-sheet content (45.0%) was observed for ZnSO_4_-WPIF. Fe^3+^ salts, particularly FeCl_3_, drastically reduced the β-sheet content to 7.2%, indicating the inhibition of ordered fibril formation. Under the same cationic conditions, SO_4_^2−^ generally yielded higher β-sheet content than Cl^−^. This increase aligns with the characteristic high β-sheet content of amyloid-like fibrils and corresponds to increased thioflavin T (ThT) fluorescence intensity, collectively indicating salt-promoted fibrillogenesis. These findings support those of prior studies on protein nanofibril formation [44]. Notably, under the same cationic conditions, the presence of sulfate anions results in a higher β-sheet content. Moreover, iron-containing salts markedly suppressed β-sheet formation. The β-sheet contents of FeCl_3_-WPIF and Fe_2_(SO_4_)_3_-WPIF were 7.2% and 15.5%, respectively. These values decreased by 7.2% and 13.2%, respectively, compared with those of the native WPIF. The stronger inhibitory effect of FeCl_3_ versus Fe_2_(SO_4_)_3_ highlights the synergistic role of Cl^−^ in impeding fibril assembly. Trivalent Fe^3+^ exhibits strong electrostatic attraction to negatively charged amino acid residues (aspartic acid, glutamic acid) in WPI, causing rapid protein aggregation and precipitation instead of ordered fibrillation. Additionally, Fe^3+^ acts as a strong Lewis acid, significantly lowering the solution pH and accelerating non-specific protein hydrolysis, which disrupts the nucleation and elongation of nanofibrils. Notably, SO_4_^2−^ can form weak complexes with free Fe^3+^, reducing its effective concentration and partially mitigating the inhibitory effect, as reflected in the higher fibril yield of the Fe_2_(SO_4_)_3_ group compared with the FeCl_3_ group. The secondary structure changes in Table 3 are highly consistent with CD spectra (Figure 6a) and ThT fluorescence results (Figure 3). The increase in β-sheet content observed in Table 3 corresponds to the blueshift and intensity changes in the CD characteristic peak near 218 nm, and also matches the higher ThT fluorescence intensity (Figure 3), since ThT specifically binds to ordered cross-β structures in fibrils. In contrast, the sharp decreases in β-sheetcontents for FeCl_3_-WPIF and Fe_2_(SO_4_)_3_-WPIF in Table 3 correspond to the loss of typical CD β-sheet signals and very low ThT fluorescence, confirming the inhibition of ordered fibril formation.

The structural characteristics of the tested WPI were analyzed using X-ray diffraction (XRD) (Figure 7b). The corresponding XRD patterns are shown in Figure 6b. All the samples exhibited diffuse broad peaks characteristic of amorphous structures [44]. Native WPIF displayed two broad diffraction peaks near 2θ ≈ 19.20°, matching the β-sheet characteristic peak at 2θ = 18.9° reported in the previous literature [31,45]. Upon the addition of NaCl and Na_2_SO_4_, the fibril aggregates exhibited broad peaks at 2θ = 20.29° and 19.28°, respectively. When KCl and K_2_SO_4_ were added, the fibril aggregates showed broad peaks at 2θ = 19.46° and 20.13°, respectively. The presence of peaks at 19.28° and 19.81° corresponded to ZnCl_2_-WPIF and ZnSO_4_-WPIF, respectively. FeCl_3_-WPIF and Fe_2_(SO_4_)_3_-WPIF have peaks at 2θ = 19.34° and 19.24°, respectively. The consistent appearance of peaks within the range of 18.67–22° corresponds to β-strand spacing in the fibrillar architecture [36]. These systematic peak position variations demonstrate distinct structural modifications induced via different salt treatments.

The above results reveal a clear mechanism via which anions and cations synergistically regulate WPI fibrillation. At low ionic strength, anions play a deterministic role in regulating nucleation kinetics and fibril morphology. SO_4_^2−^, as a kosmotropic anion, strengthens the hydration shell of protein molecules, promotes rapid nucleation, and induces the formation of short, clustered fibrils with high β-sheet content. In contrast, Cl^−^ allows sufficient fibril elongation to form long, linear fibrils. For cations, Na^+^, K^+^, and Zn^2+^ mainly regulate electrostatic interactions and reduce intermolecular repulsion, thereby promoting ordered fibrillation. Trivalent Fe^3+^ strongly binds to negatively charged amino acid residues, reduces pH, accelerates non-specific hydrolysis, and disrupts ordered assembly. The complexation between SO_4_^2−^ and Fe^3+^ weakens the inhibitory effect of free Fe^3+^, which explains the partial recovery of fibrillation in the Fe_2_(SO_4_)_3_ group. This ion-specific regulatory mechanism provides a theoretical basis for precisely tailoring the structure and functionality of protein nanofibrils in food systems. In future studies, researchers could explore the concentration-dependent effects of anions and cations, verify molecular interactions via molecular dynamics simulation, and evaluate the application performance of anion-tailored fibrils in emulsions, encapsulation, and gel systems.

## 4. Conclusions

In this study, we demonstrate that at low salt concentration strength (10 mmol/L), anion identity (Cl^−^ vs. SO_4_^2−^) plays a deterministic role in regulating the fibrillation kinetics, secondary structure, and morphology of WPI nanofibrils, with SO_4_^2−^ consistently inducing shorter, clustered fibrils and stronger kinetic suppression than Cl^−^ under the same cationic conditions. Monovalent (Na^+^, K^+^) and divalent (Zn^2+^) cations generally enhanced fibrillization (increased β-sheet content and ThT fluorescence), whereas Fe^3+^ severely inhibited fibril formation, though SO_4_^2−^ partially mitigated this inhibition. Quantitative kinetic fitting revealed that SO_4_^2−^ dramatically shortened the lag phase, indicating accelerated nucleation. XPS and XRD confirmed direct ion incorporation into fibrils and preserved β-strand spacing. This io-specific regulatory mechanism provides a theoretical basis for precisely tailoring the structure and functionality of protein nanofibrils in food systems. In future studies, researchers should elucidate the molecular mechanism of anion-specific binding, explore the reversibility and concentration dependence of Fe^3+^ inhibition, test the generalizability to other food proteins, and evaluate the functional performance of morphologically distinct fibrils in real food applications (e.g., emulsion stabilizers, encapsulation matrices).

## Figures and Tables

**Table 1:** Preparations of different samples.

Serial Number	1	2	3	4	5	6	7	8	9
Salt ions	——	NaCl	Na_2_SO_4_	KCl	K_2_SO_4_	ZnCl_2_	ZnSO_4_	FeCl_3_	Fe_2_(SO_4_)_3_
Heating 0 h	WPI	NaCl-WPI	Na_2_SO_4_-WPI	KCl-WPI	K_2_SO_4_-WPI	ZnCl_2_-WPI	ZnSO_4_-WPI	FeCl_3_-WPI	Fe_2_(SO_4_)_3_-WPI
Heating 10 h	WPIF	NaCl-WPIF	Na_2_SO_4_-WPIF	KCl-WPIF	K_2_SO_4_-WPIF	ZnCl_2_-WPIF	ZnSO_4_-WPIF	FeCl_3_-WPIF	Fe_2_(SO_4_)_3_-WPIF

**Table 2:** The fibrillation kinetic parameters of whey protein isolate (WPI) solutions with different salts after heating at 90 °C.

Samples	t_lag_	t_1/2max_	(df/dt)_max_	f_max_	Adjusted R^2^
Native WPI	2.308	5.249	0.102	888.92 ± 7.818 ^d^	0.991
NaCl-WPI	2.465	5.080	0.129	981.173 ± 15.088 ^b^	0.984
Na_2_SO_4_-WPI	2.143	5.000	0.106	975.202 ± 19.858 ^c^	0.985
KCl-WPI	1.899	4.135	0.186	962.166 ± 0.954 ^a^	0.992
K_2_SO_4_-WPI	1.773	4.121	0.167	984.862 ± 14.721 ^e^	0.993
ZnCl_2_-WPI	0.009	3.841	0.022	974.354 ± 4.386 ^f^	0.996
ZnSO_4_-WPI	0.001	3.791	0.018	984.669 ± 11.832 ^e^	0.995
FeCl_3_-WPI	3.972	5.228	0.693	173.449 ± 3.828 ^g^	0.857
Fe_2_(SO_4_)_3_	1.414	3.484	0.221	445.136 ± 1.215 ^f^	0.989

Note: t_lag_: lag time (h); t_1/2max_: time to half-maximal fluorescence (h); (df/dt)_max_: maximum fluorescence growth rate (A.U./h); f_max_: maximum ThT fluorescence intensity (A.U.). Data are presented as mean ± standard deviation. Different lowercase letters indicate significant differences (*p* < 0.05). WPI: whey protein isolate; ThT: thioflavin T.

**Table 3:** Estimations of secondary structure content of native WPI, native WPIF, NaCl-WPIF, Na_2_SO_4_-WPIF, KCl-WPIF, K_2_SO_4_-WPIF, ZnCl_2_-WPIF, ZnSO_4_-WPIF, FeCl_3_-WPIF, and Fe_2_(SO_4_)_3_-WPIF.

Samples	α-Helix (%)	β-Sheet (%)	β-Turn (%)	Random Coil (%)
Native WPI	55.00%	28.70%	19.30%	1.70%
Native WPIF	16.00%	37.10%	20.20%	32.20%
NaCl-WPIF	7.30%	43.60%	19.60%	31.90%
Na_2_SO_4_-WPI	7.70%	40.20%	20.70%	33.10%
KCl-WPIF	9.20%	38.30%	22.20%	33.00%
K_2_SO_4_-WPIF	12.90%	42.90%	21.10%	31.90%
ZnCl_2_-WPIF	12.90%	42.90%	21.10%	31.90%
ZnSO_4_-WPIF	7.10%	45.00%	19.00%	31.50%
FeCl_3_-WPIF	43.20%	7.20%	19.10%	35.30%
Fe_2_(SO_4_)_3_-WPIF	37.40%	15.50%	20.80%	35.30%

Note: Secondary structure contents (α-helix, β-sheet, β-turn, random coil) calculated from far-UV CD spectra (190–260 nm). The characteristic CD peaks for each structure are identified in the text.

## Data Availability

The original contributions presented in the study are included in the article; further inquiries can be directed to the corresponding authors.

## References

[1] Lin Y., Roos Y.H., Miao S. (2025). The effect of whey/soy protein fibril systems on the properties of fish gelatin stabilized emulsion gels. Food Hydrocoll..

[2] Peng Y., Wang C., Yu J., Wu J., Wang F., Liu Y., Li X. (2023). Self-assembly mechanism of rice glutelin amyloid fibril aggregates obtained through experimental and molecular dynamics simulation analysis. Food Hydrocoll..

[3] Teng J., Xu C., Lu Z., Li T. (2025). Different types of anions mediated the formation of rice glutelin fibrils: Aggregation behaviors and structural characteristics. Food Chem..

[4] Kazemi-Taskooh Z., Varidi M. (2025). A mechanistic insight into whey protein isolate (WPI) fibrillation driven by divalent cations. Food Chem..

[5] Qi X., Li Y., Shen M., Yu Q., Chen Y., Xie J. (2024). Formation of rice protein fibrils is highly sensitive to the different types of metal ions: Aggregation behavior and possible mechanisms. Food Chem..

[6] Yang X., Song Y., Guo R., Xu H., Jin C. (2024). Structural modification of whey protein nanofibrils by a multiround induction pathway for enhancing the stability of Pickering emulsions. Food Hydrocoll..

[7] Cao Y., Adamcik J., Diener M., Kumita J.R., Mezzenga R. (2021). Different folding states from the same protein sequence determine reversible vs irreversible amyloid fate. J. Am. Chem. Soc..

[8] Loveday S.M., Wang X.L., Rao M.A., Anema S.G., Creamer L.K., Singh H. (2010). Tuning the properties of β-lactoglobulin nanofibrils with pH, NaCl and CaCl2. Int. Dairy J..

[9] Li T., Wang D., Zhang X., Chen Z., Wang L. (2024). Specific ions effect on aggregation behaviors and structural changes of amyloid fibrils from rice glutelin. Food Chem..

[10] Mijin N., Milošević J., Stevanović S., Petrović P., Lolić A., Urbic T., Polović N. (2023). Amyloid-like aggregation influenced by lead(II) and cadmium(II) ions in hen egg white ovalbumin. Food Hydrocoll..

[11] Kang B., Tang H., Zhao Z., Song S. (2020). Hofmeister series: Insights of ion specificity from amphiphilic assembly and interface property. ACS Omega.

[12] Cao Y., Mezzenga R. (2019). Food protein amyloid fibrils: Origin, structure, formation, characterization, applications and health implications. Adv. Colloid Interface Sci..

[13] Hoppenreijs L.J.G., Fitzner L., Ruhmlieb T., Heyn T.R., Schild K., van der Goot A.J., Boom R.M., Steffen-Heins A., Schwarz K., Keppler J.K. (2022). Engineering amyloid and amyloid-like morphologies of β-lactoglobulin. Food Hydrocoll..

[14] Loveday S.M., Su J., Rao M.A., Anema S.G., Singh H. (2011). Effect of calcium on the morphology and functionality of whey protein nanofibrils. Biomacromolecules.

[15] Li T., Wang L., Zhang X., Yu P., Chen Z. (2021). Effect of ionic strength on assembly behaviors and rheological properties of rice glutelin based fibrils. J. Cereal Sci..

[16] Ji F., Xu J., Ouyang Y., Mu D., Li X., Luo S., Shen Y., Zheng Z. (2021). Effects of NaCl concentration and temperature on fibrillation, structure, and functional properties of soy protein isolate fibril dispersions. LWT.

[17] Loveday S.M., Su J., Rao M.A., Anema S.G., Singh H. (2012). Whey protein nanofibrils: Kinetic, rheological and morphological effects of group IA and IIA cations. Int. Dairy J..

[18] Miao L., Zhu J., Peng X., Feng J., Dong H., Tong X., Wang H., Jiang L. (2023). Effects of CaCl2 concentration on fibrils formation and characteristics of soybean protein isolate and β-conglycinin/glycinin. Food Hydrocoll..

[19] Wang D., Zhang C., Ye J., Chen M., Zhou J., Li T., Li J., Zhang X., Wang L. (2025). Hofmeister and electrostatic modulation of the structure and polymorphism of rice protein fibrils. Food Chem..

[20] Cheng C., Xu Q., Li Y., Haubruge E., Liao N., Zhu G., Liu K. (2026). Preparations, characterizations, assembly behaviors, applications, and perspectives of whey protein amyloid fibrils: A review. Food Chem..

[21] Poniková S., Antošová A., Demjén E., Sedláková D., Marek J., Varhač R., Gažová Z., Sedlák E. (2015). Lysozyme stability and amyloid fibrillization dependence on Hofmeister anions in acidic pH. J. Biol. Inorg. Chem..

[22] Wawer J., Szociński M., Olszewski M., Piątek R., Naczk M., Krakowiak J. (2019). Influence of the ionic strength on the amyloid fibrillogenesis of hen egg white lysozyme. Int. J. Biol. Macromol..

[23] Liang Y., Zheng C., Yang L., Huang M., Liu J., Liu H., He B., Wang J. (2025). Investigation of the effect of NaCl concentrations on the formation of amyloid fibrils during the cooking of wheat noodles. Foods.

[24] Xu J., Zhao X., Ban Q. (2025). Ultrasound-assisted fibril formation enhances complexation of oat globulin with quercetin: Mechanism, structure evolution, delivery performance. Foods.

[25] Yang B., Tang Y., Xu T., Dai S., Fang Q., Lv G., Wang H., Jiang L. (2026). The stability and digestive characteristics of soybean protein fibril/κ-carrageenan composite gels for riboflavin encapsulation. Foods.

[26] Rathod G., Amamcharla J. (2024). Milk whey protein fibrils—Effect of stirring and heating time. Foods.

[27] Zhang Q., Deng Y., Lu Y., Han L., Ma Q., Guo L., Fan F. (2025). Effects of treatment methods on the formation, structure, and functional properties of soy protein amyloid fibrils. Foods.

[28] Figueiredo M., Sárkány Z., Rocha F., Martins P.M. (2025). Challenges and advances in the encapsulation of bioactive ingredients using whey proteins. Foods.

[29] Ye X., Capezza A.J., Xiao X., Lendel C., Hedenqvist M.S., Kessler V.G., Olsson R.T. (2021). Protein nanofibrils and their hydrogel formation with metal ions. ACS Nano.

[30] Morris A.M., Watzky M.A., Agar J.N., Finke R.G. (2008). Fitting neurological protein aggregation kinetic data via a 2-step, minimal/”ockham’s razor” model:  The Finke−Watzky mechanism of nucleation followed by autocatalytic surface growth. Biochemistry.

[31] Yang X., Xie M., Guan C., Guo R., Ma C., Xu H., Shao M. (2022). Effect of CaCl2 on 2 heat-induced whey protein concentrate fibrillation pathways: Spontaneous and nuclear induction. J. Dairy Sci..

[32] Feng Z., Li L., Zhang Y., Li X., Liu C., Jiang B., Xu J., Sun Z. (2019). Formation of whey protein isolate nanofibrils by endoproteinase GluC and their emulsifying properties. Food Hydrocoll..

[33] Shan G., Xu Z., Jiang L., Zhang Y., Sui X. (2024). Fabrication and characterization of glycerin-plasticized soy protein amyloid fibril scaffolds by unidirectional freeze casting method. Food Hydrocoll..

[34] Yang X., Guan C., Ma C., Xu H. (2023). Nuclei-induced formation of amyloid fibrils in whey protein: Effects of enzyme hydrolysis on the ability of nuclei to induce fibril formation. Food Chem..

[35] Liu C., Wang Y., Dai X., Zhang Y., Yang Y., Jiang B., Li D., Feng Z. (2024). Post-self-assemble of whey protein isolation nanofibrils and its contribution to the stability of pickering emulsion. Food Hydrocoll..

[36] Guan C., Bing S., Yang X., Guo R., Chen Y., Xu H., Yu G. (2022). Homogeneous nuclei-induced, secondary nuclei-induced, and spontaneous whey protein concentrate nanofibril formation through different pathways. J. Dairy Sci..

[37] Acharya V.V., Chaudhuri P. (2021). Modalities of protein denaturation and nature of denaturants. Int. J. Pharm. Sci. Rev. Res..

[38] Afkhami R., Varidi M.J., Varidi M., Hadizadeh F. (2023). Improvement of heat-induced nanofibrils formation of soy protein isolate through NaCl and microwave. Food Hydrocoll..

[39] Wang X., Liang X., Zhao J., Cao Z., Zhang Y., Jiang L., Xu Z., Sui X. (2025). Acceleration of soy protein amyloid fibrils formation: Homologous seeding mechanism. Food Chem..

[40] Loveday S.M., Wang X.L., Rao M.A., Anema S.G., Singh H. (2011). Effect of pH, NaCl, CaCl2 and temperature on self-assembly of β-lactoglobulin into nanofibrils: A central composite design study. J. Agric. Food Chem..

[41] Cao Z., Wang X., Zhao J., Liang X., Zhang Y., Jiang L., Xu Z., Sui X. (2024). Elucidating the modulatory influence of Hofmeister divalent ions on the structural dynamics and rheological properties of soy protein amyloid fibrils. Food Hydrocoll..

[42] Chang C., Li X., Li J., Su Y., Gu L., Xiong W., Yang Y. (2023). Fabrication mechanism and functional properties of ovalbumin fibrils prepared by acidic heat treatment. J. Sci. Food Agric..

[43] Li T., Zhou J., Wu Q., Zhang X., Chen Z., Wang L. (2023). Modifying functional properties of food amyloid-based nanostructures from rice glutelin. Food Chem..

[44] Wei Z., Huang Q. (2019). Assembly of iron-bound ovotransferrin amyloid fibrils. Food Hydrocoll..

[45] Kumar E.K., Haque N., Prabhu N.P. (2017). Kinetics of protein fibril formation: Methods and mechanisms. Int. J. Biol. Macromol..

